# Axillary Paget disease with a visible satellite: a case report and literature review

**DOI:** 10.1186/s13000-021-01131-1

**Published:** 2021-08-01

**Authors:** Wu-Yang Ji, Bin Luo, Xue-Wei Wang, Ying Xiao, Jin-Yi Tian

**Affiliations:** 1 Department of General Surgery, Beijing Tsinghua Changgung Hospital, School of Clinical Medicine, Tsinghua University, No.168 Litang Road, Changping District, Beijing, 102218 China; 2 Department of Pathology, Beijing Tsinghua Changgung Hospital, School of Clinical Medicine, Tsinghua University, No.168 Litang Road, Changping District, Beijing, 102218 China

**Keywords:** Extramammry Paget disease, Satellite Paget lesion, Wide local excision, Shoulder motor function

## Abstract

**Background:**

Extramammary Paget disease (EMPD) is an uncommon malignancy affecting apocrine gland–bearing skin, such as vulvar, perianal, axillary and penoscrotal areas. Paget cells are sometimes detected outside clinical border in a phenomenon called subclinical extension. Satellite is one of the patterns of subclinical extension which is likely to be invisible. The standard management strategy for EMPD without distant metastasis is a complete surgical removal, sometimes called wide local excision. However, there is no consensus regarding surgical margin width to decrease the high recurrence rate. Here we describe the first macroscopically visible satellite of axillary EMPD and wide local excision of both main lesion and the satellite lesion with only 0.5 cm margin, succeeded by a short review of the literature.

**Case presentation:**

A 48-year-old female with a red macule in the right axilla was presented to our clinic. A well-demarcated 4 cm × 3 cm erythematous plaque was observed in the right axilla, and a similar lesion measuring 0.5 cm × 0.3 cm was found 3.5 cm away from the primary site. Breast and axillary node examination was unremarkable. Biopsy of the large plaque revealed Paget disease, then we performed a local extended excision of both lesions with a 0.5 cm margin, all margins negative indicated, by frozen pathology. Pathology revealed the nature of the satellite beside the main lesion also as Paget disease. The patient is currently followed-up for 20 months and has shown no signs of recurrence, with normal shoulder motor function.

**Conclusion:**

We have report the first visible satellite of extramammary Paget disease, indicating the necessity of an extended local resection of both the main leision and the satellite lesion. Considering the anatomical structure of axillary Paget disease, a 0.5 cm negative surgical margin indicated by frozen pathology might be sufficient to sustain the shoulder motor function.

## Case report

A 48-year-old female consulted our general surgery clinic due to a red macule in her right axilla; the macule had gradually enlarged from 1 cm × 1 cm to 4 cm × 3 cm in 4 years. The lesion repeatedly ulcerated and healed without itching or pain. The patient had not previously sought clinical treatment for the lesion. During its progression, she did not notice any breast masses, nipple discharge or skin changes in the nipple areola or anogenital area. She also denied having any other systemic symptoms or history of malignancy. Her previous history included the presence of a uterine fibroid for 10 years and a hysteroscopic endometrial polypectomy performed 1 year prior. Clinically, a well-demarcated 4 cm × 3 cm erythematous plaque was observed in the right axilla. A similar lesion measuring 0.5 cm × 0.3 cm was found 3.5 cm away from the primary plaque. Axillary nodes were not palpable on either side. Breast examination was unremarkable.

A biopsy of the large axillary plaque was performed, and Paget disease was diagnosed, characterized by the presence of Paget cells—large round cells with abundant pale or granular/dusty cytoplasm, pleomorphic vesicular nuclei and prominent nucleoli. Immunohistochemistry showed that Paget cells were diffusely positive for P63, CK7, and CK19 and negative for S-100.

**Fig. 1:**
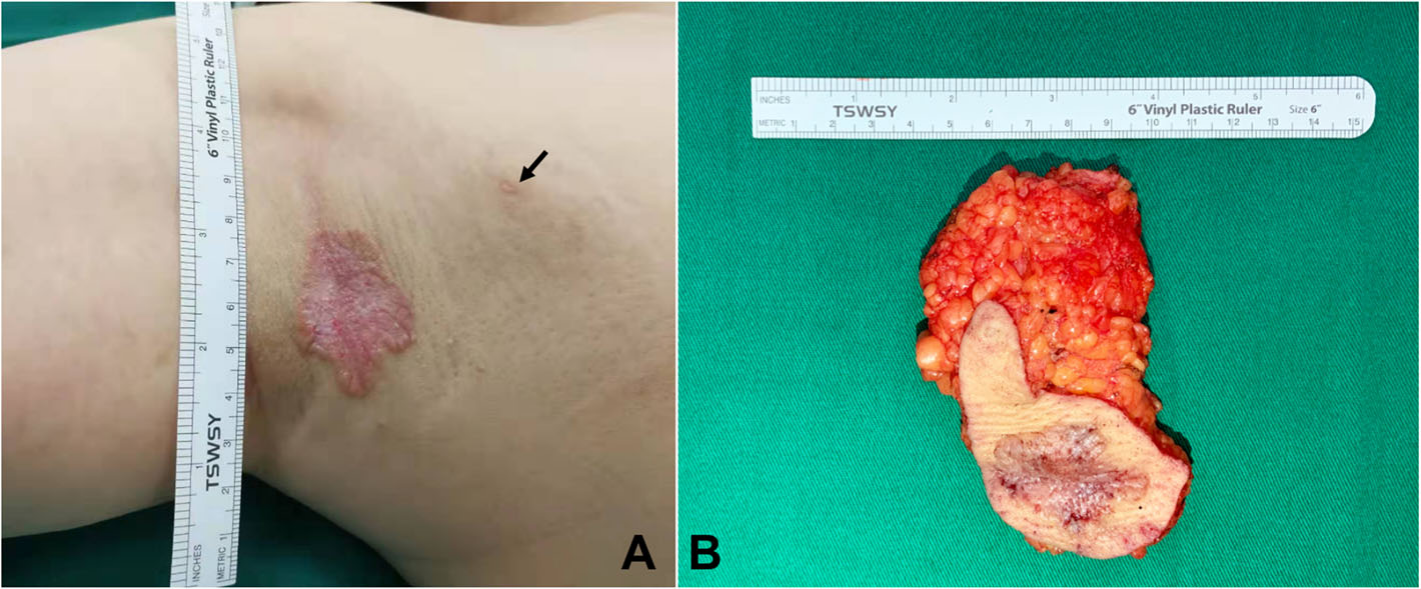
Axillary skin lesions and wide local excision sample. **A** The main lesion in the right axilla measured 4 cm × 3 cm and consisted of a well-demarcated erythematous plaque. Another hypopigmented erythematous plaque, measuring 0.5 cm × 0.3 cm, was located 3.5 cm away from the lesion (black arrow). **B** Wide local excision of the main lesion and the satellite lesion (black arrow) with a margin of 0.5 cm to 1 cm and the accessory breast tissue

**Fig. 2:**
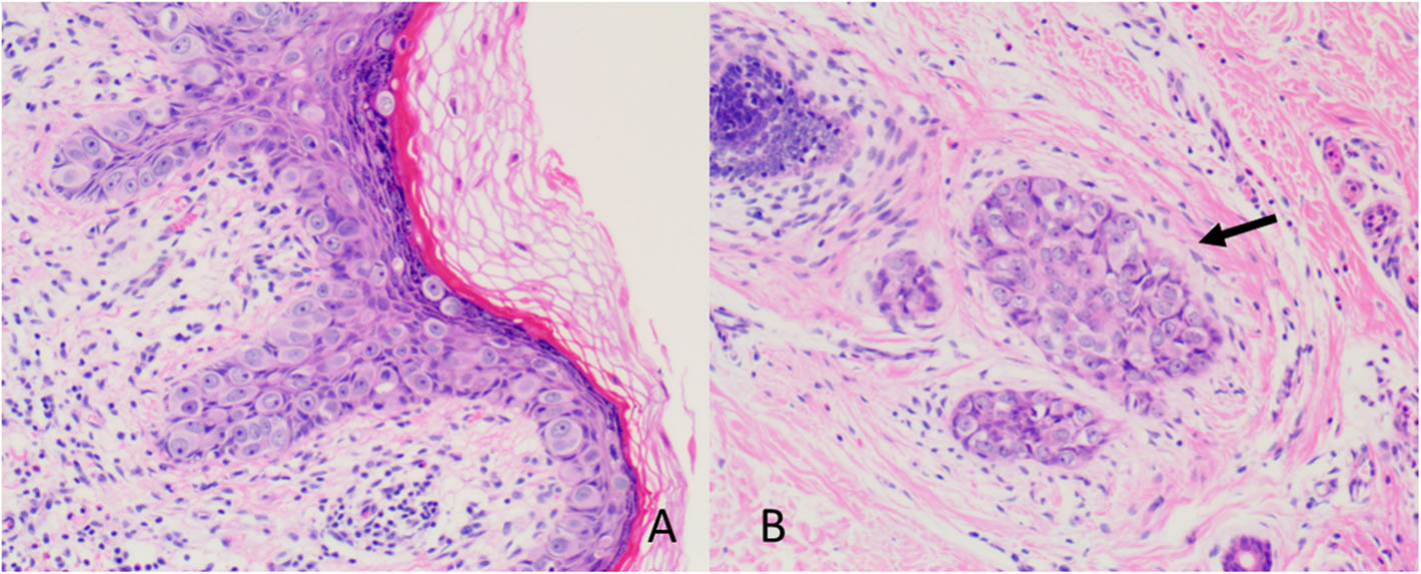
**A** Pathology of the lesion showed Paget disease involving the epithelium of the skin (10× magnification); **B** Focal dermal invasion of Paget cells (black arrow) (100× magnification)

**Fig. 3:**
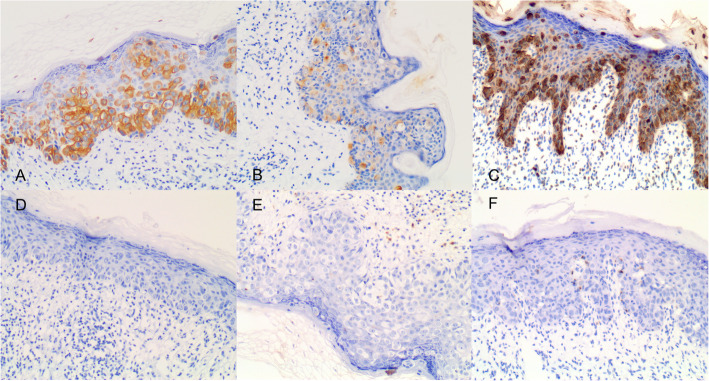
Immunohistochemical staining of the axillary Paget disease lesion (100× magnification). **A** Positive staining for CK7; **B** Weakly positive staining for GCDFP15; **C** Positive staining for CEA; **D** Negative staining for CK20; **E** Negative staining for S100; **F** Negative staining for HMB45

We performed a local extended excision of both lesions with negative margins by frozen section (Fig. 1). The final pathology results revealed that the two plaques were both Paget disease. Paget cells were arranged in small clusters and occupied the whole thickness of the overlying epidermis measured as carcinoma in situ, which was hyperkeratotic. There was tumor extension into the epithelium of the skin, consistent with a pattern of pagetoid spread, with focal dermal invasion (Fig. 2). Supplemental immunohistochemistry showed weakly positive staining for gross cycstic disease fluid protein 15(GCDFP15), diffuse positive staining for Muc-1 and negative staining for CK20 (Fig. 3).

The patient is currently at 20 months of follow-up and has shown no signs of recurrence.

## Discussion

Paget disease is a slow-growing intraepidermal adenocarcinoma first described at the nipple by Paget in 1874; in this location, it is known as mammary Paget disease. In 1889, the first extramammary Paget disease (EMPD) of the scrotum and penis was identified; EMPD is an uncommon malignancy accounting for 6.5% of all cases of Paget disease and affecting apocrine gland–bearing skin, such as the vulvar, perianal, axillary and penoscrotal areas [1]. It most commonly affects patients over 65 years of age, although patients under the age of 50 have been described [2, 3]. The incidence is 10/10^6^ and 0.9/10^6^ in Asian and Western populations, respectively [4], and women are affected 3 times more commonly than men [5]. Clinically, EMPD often presents as a scaly superficial pigmented macule, mimicking a melanocytic lesion. Histological examination typically reveals carcinoma in situ with an increased number of melanocytes scattered between the Paget cells [6]. It is believed that up to 42% of patients with EMPD may have an underlying primary or noncutaneous malignancy [7]. Axillary lesions may be associated with breast malignancy. A few histological markers are useful for confirming the diagnosis of EMPD. Standard hematoxylin and eosin staining shows the presence of large cells with a bluish-tinted cytoplasm, called Paget cells [8], and periodic acid-Schiff and Alcian blue staining are also positive. Regarding immunohistochemistry, positive staining for CK7, CEA, CAM5.2 and GCDFP15 [9] and negative staining for S100, CK20 and melanocytic markers [10] are useful for Paget diagnosis.

We have two special matters to discuss. First, Paget cells are sometimes detected outside the clinical border in a phenomenon called subclinical extension. In EMPD, there are at least two patterns of subclinical extension: continuous and satellite lesions [10]. Subclinically extended Paget cells in small foci are likely to be invisible and are detected outside hypopigmented patches with erythema, whereas small satellite lesions are found only in the vicinity of the main lesions [11]. The spreading distance of subclinical extension is still controversial, ranging from several millimeters to several centimeters according to reports [12–15]. Paget cells might migrate in the epidermis from the border of the main lesion and proliferate as a separate satellite lesion.

In our case, a visible lesion located 3 cm away from the large main lesion, which was pathologically proven to be a Paget disease satellite lesion. Visible satellites located outside the main lesion are easily misdiagnosed. Considering the possibility of epidermal extension from the main lesion to the satellite, it was necessary to perform a total resection of the main lesion and satellite lesion along with the suspected extension pathway. With careful incision design, the scar was hidden in the axilla to optimize the aesthetics. This is the first described subclinical extension of axillary EMPD to a macroscopically visible satellite more than 1 cm away, and we report the wide local excision of both lesions.

The second special matter in our case is the balance of margin safety and conservation of axillary mobility. The standard management strategy for EMPD without distant metastasis is complete surgical removal, sometimes called wide local excision. However, regardless of the surgical approach, a high recurrence rate of 30–60% has been reported [16], likely due to clinically ill-defined margins, microscopic extension of tumor cells, and multifocal disease. The term “wide local excision” has not been well defined in the EMPD literature, as there is no agreement on margin width. Some data suggest that a margin of 1 cm is sufficient for lesions with clinically distinct margins [17], whereas other reports advocate for safety margins extending 3 to 5 cm past the tumor circumference on all sides [18, 19]. However, in a retrospective study of 66 patients, there was no significant difference in recurrence rate between patients in the ≤2-cm and > 2-cm margin excision groups, suggesting that a 2-cm margin is sufficient for treating EMPD [20]. Moreover, Murata retrospectively reviewed 46 EMPD patients who had been treated with 1-cm margin excision and found no local recurrence in several years of follow-up. The microscopically observed gap between the histopathological and clinical borders measured less than 2 mm. As determined by mapping biopsy and two-photon microscopy, the prevalence of subclinical extension greater than 1 cm was less than 10% 12 [11, 21]. Based on these studies, the latest guidelines of the Japanese Skin Cancer Society recommend predetermined surgical margins of 1 cm for clinically well-demarcated EMPD [22].

However, all studies and guidelines on margin discussion are mainly based on anogenital EMPD which is different from the axillary EMPD considering joint motion and esthetic issues. There are no large retrospective reviews or guidelines for axillary EMPD to date. Both of hip joints and shoulder joints are ball-and-socket joints, but hip joints are weight-bearing, has small articular caput and deep glenoid cavity, enabling the high stability and limited range-of-motion (ROM). However, the shoulder joints need higher ROM, excessive wide margin excision might affect the shoulder joints distinctly, especially the abduction motion which plays a key role in upper limb function. This is, however, not the case as flexion and extension motions suffice fundamental functions for hip joints. Furthermore, axilla is more exposed in daily life than anogenital area, we should take the shoulder joint motion, the oncological safety, and the esthetic purposes into axillary EMPD surgical consideration. It’s difficult to ensure the 1 cm margin resection because of the axillary anatomical structure. Considering the well correspondence between the clinically border and the histopathology border, it might be acceptable for axilla Paget disease to diminish the margin to 0.5 cm for the well-demarcated lesion, with the precisely drawing encircling margin line of the lesion before the surgery and the multiple margin sampling on 3, 6, 9, 12 o’clock of the residual cavity to receive fast frozen pathology during the surgery. We believe the 3, 6, 9, 12 o’clock margin-free result has high predictive value of the margin with H&E staining only. It’s clear to distinguish Paget cells without further immunohistochemical staining. Because multiple sampling fast frozen pathology can be very time-consuming during the surgery, it is hard to fulfill the Morh surgery in governmental hospitals. However, large cohort studies and long-term follow-up are still needed.

In conclusion, we should carefully check whether there is any satellite extension around the main lesion during the treatment of axillary EMPD. The standard of recommended wide local excision is not clear in axillary EMPD; the 1 cm excision margin is not feasible given the anatomical structure of the axilla, and 0.5 cm is probably acceptable when combined with frozen pathology guidance. Further clinical experiences are still needed regarding the excision margin and prognosis in axillary Paget disease.

## Data Availability

The datasets used and/or analyzed during the current study are available from the corresponding author on reasonable request.

## Abbreviations

EMPD: Extramammary Paget disease
GCDFP15: Gross cycstic disease fluid protein 15
